# Widespread Dermatophytosis in a Patient Treated for Leprosy Type II Reactional State after MDT/WHO-MB Release

**DOI:** 10.4269/ajtmh.18-0280

**Published:** 2018-10

**Authors:** Felipe Tavares Rodrigues, Marcos Roberto Pereira Cardozo, José Augusto da Costa Nery

**Affiliations:** 1 Escola de Medicina e Cirurgia do Rio de Janeiro—Universidade Federal do Estado do Rio de Janeiro—UNIRIO, Rio de Janeiro - RJ, Brazil;; 2 Ambulatório Souza-Araújo, Instituto Oswaldo Cruz, Fundação Oswaldo Cruz—FIOCRUZ, Rio de Janeiro - RJ, Brazil

A 23 year-old male with no immunosuppressive diseases, including Cushing syndrome visited Souza Araújo Ambulatory, Fundação Oswaldo Cruz, Reference Center, Brazil, for treatment of leprosy, 3 years after a 12-month standard treatment of multibacillary lepromatous leprosy. He presented with a residual 3+ bacilloscopy index, ulnar nerve neuritis, and an exuberant erythema nodosum leprosum (ENL) reaction, which was not controlled with a previous corticosteroid therapy regimen. No signs of fungal infection were found this time. Therefore, thalidomide regimen was administered at a dose of 200 mg/day with 40 mg/day prednisone; both drugs were gradually reduced at monthly intervals during medical visits, and the treatment was stopped after 3 months. However, 4 months later, the patient presented with new skin lesions (Figure 1A–C), suggesting that ENL had relapsed. Culture samples of the epithelium and nails showed the presence of *Trichophyton mentagrophytes* and *Trichophyton tonsurans* (Figure 1D and E). In addition, a skin biopsy showed histopathological features of leprosy activity (logarithmic index of biopsies: 3.8). The recommended treatment was to restart the rifampin, dapsone and clofazimine 12-month WHO/MDT MB scheme after dermatophytosis resolution with oral terbinafine administration.

**Figure 1.**
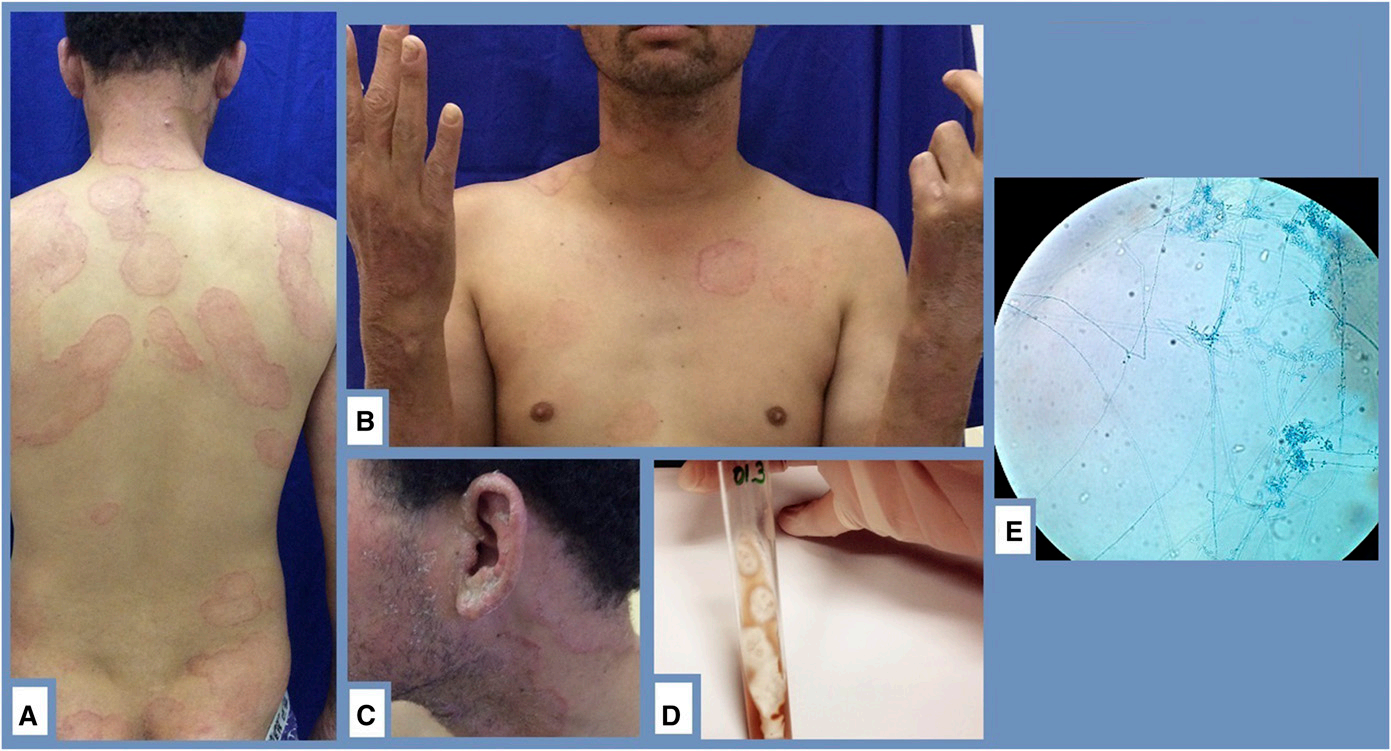
(**A**) Tinea corporis presented as a disseminated, circinate, and scaly rash with an active, erythematous, well-defined border. (**B**) The patient presented with exuberant tinea barbae lesions, in addition to nail dystrophy due to onychomycosis and an ulnar neuropathy, leading to a partial claw hand deformity of leprosy. (**C**) The patient’s earlobe exhibited an infiltration typically seen in lepromatous leprosy patients; the earlobe also developed skin peeling related to the fungal infection. (**D**) The white colonies of *Trichophyton mentagrophytes* and *Trichophyton tonsurans* showing variations in texture: granular to powdery, flat, and often with radial grooves. The growth of both dermatophytes on potato dextrose agar media for 30 days at 25°C. (**E**) Final fungal identification was based on chlamydospore-like structure identification by light microscopy from the cultured petri dishes. Microscopic morphology of *T. tonsurans* is described with numerous variably shaped microconidia formed along the septate hyphae; this feature is known as the birds on a wire pattern. Lactophenol blue stain was used, ×250 magnification. This figure appears in color at www.ajtmh.org.

Globally, Brazil ranks at the top in terms of the number of new leprosy cases. Erythema nodosum leprosum, a leprosy reactional state, has a tendency to occur in multibacillary patients, mainly in those with a lepromatous pole. In most cases, immunosuppressive drugs are necessary to control the reaction, leading to an environment conducive to opportunistic infections, including fungal infections.^1^

Dermatophytes are fungi causing superficial skin infections; they feed on the keratin present in the corneum skin. *Trichophyton mentagrophytes* are anamorphic species, that is, they are asexual or imperfect, besides being antrophilic and zoophilic. They are also found in dogs, rodents, and rabbits.^2^ Furthermore, *T. tonsurans* are anantrophilic species, more commonly seen in infants and mainly affecting the scalp area; occurrence of this pathogen is also related to poverty.^3^

This article highlights the importance of dermatophytosis as a late potential iatrogenic effect of corticosteroid therapy; in addition, antifungal immune lymphocyte response in lepromatous leprosy pole is impaired due to Th2/Treg modulation.^4^ Furthermore, clofazimine can also play a role in dermatophyte dissemination via ichthyosis, its recognized adverse effect, which may also favor fungal proliferation.

Although possible, the association between leprosy and widespread dermatophytosis has rarely been described in the literature^5^; dermatophytosis can be often misdiagnosed by inexperienced physicians as syphilitic roseola, leprosy reactional states, such as erythema multiforme, or a leprosy relapse; therefore, histopathological and microbiological investigations must be encouraged, whenever possible.

## References

[1] Kaur I Dogra S Narang T De D, 2009 Comparative efficacy of thalidomide and prednisolone in the treatment of moderate to severe erythema nodosum leprosum: a randomized study. Aust J Derm 50: 181–185. 10.1111/j.1440-0960.2009.00534.x 19659979

[2] Weitzman I Summerbell R, 1995 The dermatophytes. Clin Microbiol Rev 8: 240–259. 7621400 10.1128/cmr.8.2.240 PMC172857

[3] Salci TP Salci MA Marcon SS Salineiro PHB Svidzinski TIE, 2011 *Trichophyton tonsurans* in a family microepidemic. An Bras Dermatol 86: 1003–1006. 22147044 10.1590/s0365-05962011000500022

[4] Sousa JR Sotto MN Quaresma JAS, 2017 Leprosy as a complex infection: breakdown of the Th1 and Th2 immune paradigm in the immunopathogenesis of the disease. Front Immunol 8: 1635. 29234318 10.3389/fimmu.2017.01635 PMC5712391

[5] Thangaraju P Giri V Singh H Kumar V Ali S, 2014 Tinea barbae: in released from treatment (RFT) Hansen’s disease patient. J Clin Diagn Res 8: YD01–YD02. 10.7860/JCDR/2014/8250.4565 PMC4149128 25177622

